# Supplementary material for: Endogenous antisense RNA curbs CD39 expression in Crohn’s disease
Source: Nat Commun. 2020 Nov 18;11:5894. doi: 10.1038/s41467-020-19692-y (PMC7676266; doi:10.1038/s41467-020-19692-y)
Supplement: Supplementary file 1 — Supplementary Information [file 41467_2020_19692_MOESM1_ESM.pdf]

Endogenous antisense RNA curbs CD39 expression in Crohn's disease

Harshe RP, Longhi MS *et al.*

Supplementary Notes

ENST00000452728.5 ENTPD1-AS1-209 (cDNA, 858 nucleotides) PCR product size: 135bp (variant 1, v1)

CTGTCATGGCTGCTGTCAGTGAGGGCCCGTAATGGAGATCGAGTCAAGGGTTGCCCAGGGACACTTAAGAGCCTTCGGCTTCAAGG
AGGCCTCTCAAAGGACAAAGGATGAGACTGCCTGGCGGCAGAGTGCCCCCCACAATCGTAGGAACTGTGTCGCCGCGCTTCCCGTT
AACTTGCCGCTAAGTTTTTCTAGAAAAGTCGCCCTTCTTGCCAGGGGTGTCCTATAGACTTTGTCATGGAGTGAAACAGAAACAAA
TACTTCATATACCCCTCATGATGTCATGAGATGACTGTTTGGATTAATCATCTTCAGACCAAGACTCTTCAGCTGGCTTGGCTTTT
TCAGAGGAAACAGAGACTCTCAGACATGAATACTGTGACACTGACCACCAGGCCAACTTAACATCAGCAGATATCCTGTCTCAGAT
GAAGAGAATGGCCTGTCCAGAAGACAGCCAAATACATCCTTCAAACAGACTTCACTGCAGGAGACAGAGTGGATTCATCCTGTATT
CTCCATGTAGACTTCCATCTTCATGAGTACTCCAAGAAGATGGTTCAGTTGACTATTGCAAAGTCTCAGGTATGTCTTTATCAGCA
GCATGAAAATGGACTAATAGTACCAGTAGAGTGGGGCATTGCTGAAAAGATACCCGAAAATGTGGAAGCAACTTTGGAACTGGGTG
TTGAAATAATCTGGAGGGCTCAGAAGAAGATAGGAAAATGTAGGAAAGTTTGGAACCTCCTAGAGACTGTGAATGGCATTGACAAA
AATACTGATAGTGATATGAACAATAAGGTCCAGGCTGAGGTGGTCTCAGATGGAGATGAAGACCTTGTTGGGAACTGAAGTAAA

ENST00000414006.2 ENTPD1-AS1-201(cDNA 2.457Kb) PCR product size: 257bp (variant 2, v2)

GGTGCGCCAAGTCTGTTCGCCGCCGGGGTCTATCACCCCCGCCCCTCGCCTCTGCCCGTGAGCGTGTGAGTTGTAGCGTACCCTGG
GGTCTGTTCTGGAGGAGGCCTGAGAGCGGGGTTGCCTGGGGCGTGGGTCTCTCATCCCTCTCGTCCTGGTGAAGAAGAAGAGTATA
CCCAGCGGCTGGCTTGGCTTTTTCAGAGGAAACAGAGACTCTCAGACATGAATACTGTGACACTGACCACCAGGCCAACTTAACAT
CAGCAGATATCCTGTCTCAGATGAAGAGATGGAGTCTCGCACTGTCGCCCCAGTTGGAGTGTAATGGTGCGATCTCGGCTCACTGC
AAGCTCCGCCTCCCGGGTTCATGCCATTCTCCTGCCTCAGCCTCCTGAGTAGCTGGAACTACAGAATGGCCTGTCCAGAAGACAGC
CAAATACATCCTTCAAACAGACTTCACTGCAGGAGACAGAGTGGATTCATCCTGTATTCTCCATGTAGACTTCCATCTTCATGAGT
ACTCCAAGAAGATGGTTCAGTTGACTATTGCAAAGTCTCAGGTATGTCTTTATCAGCAGCATGAAAATGGACTAATAGTACCAGTA
GAGTGGGGCATTGCTGAAAAGATACCCGAAAATGTGGAAGCAACTTTGGAACTGGGTGTTGAAATAATCTGGAGGGCTCAGAAGAA
GATAGGAAAATGTAGGAAAGTTTGGAACCTCCTAGAGACTGTGAATGGCATTGACAAAAATACTGATAGTGATATGAACAATAAGG
TCCAGGCTGAGGTGGTCTCAGATGGAGATGAAGACCTTGTTGGGAACTGAAGTAAAGGTGACCCTTGTTATGTTTTAGCAAGGAGA
CTGGCAGCATTTCACCCTTGCCCTAGAGATTTGTGGAACTTAGAGCTTGAGAGAGATGACTTAGGGTATCTGGTGGAATAAATTTC
TAAGCAGCAAAGCATTCAAAAGGTGACTTGGGTGCTGTTAAAAACATTCTGTTTTAAAAGGGAAACAAGAGCATAAATGTGGAAAA
TCTGCAGCCTGATGATGCAGTAGAAAAGAAAAACCCATTTTTTTTAAGGAGAAATTCAAGCTGGCTGCAGAAATTTGCATAAGCAG
CAAGGAGCCTAATGTTAATCCCCAAGACCATGGGGAAAATGTCTCCAGGTCATGTCAGAGACCTTCATGGCAGCCCCTCCCATCAC
AGGCCCAGAGGCCCAGGAGGAAAAAGTGGTTTTGTGGGCTGGGCCCAGGGTCTCTGTGCTGTGTGCAGCCTAGGGACTTGGTGCCC
TGTGTCCCAGCTGCTCCAGCCATGGCTGAAAGGAGCCAACATAGAGCTTGGGCTGTGGCTTCAGAGGGTAGAGGCCCCAAGCATTG
GCAGCTTCCATGTGATGTTGAGCCTGTGGGTGCAGAGATGTCAAGAATTGAGATTTGGGAACCTCTGCCTAGATTTCAGAATATAT
ATGGAAACACCTGGATGTCCAGGCAAAAGTTTGCTGCAGGGGCAGAGCCCTCATGGGGAACCTCTCCTAGGGCAGTGTGGAAGGGA
AATGTGGGGCAGAGCCCCCACACAGAGTCCCTGTTGGGGCACTGCCTAGTGGAGCTGTGAGAAGAAGGCCACAGTCCCAGAACGGT
AGACCCCAGAATGGTAGACCCCAGAATGGTAGATCAACCACCAACTTGCATCTTGTGCCTGGAAAAGCTACAGACACTCAACATCA
GCCTGAGAAAGCAGCCAGGAGGGAGGCTGTACTCTGCAAAGCCACAGGGGCGGAGCTGCACAAGACCATGGGAACCTATCTCTTGC
ATCAGCATGACCTGAATGTGAGACATGGAGTCAAAGGAGATCATTATGGAGCTTTAAAATTTGACTGCCCCACTGGATTTCAGACT
TGCATGGACCCTGTAACCCCTTTGTTTTGGCCAATTTCTCTCATTTGGAACAGCTGTATTTACCCAATACCTGTACCCCCCATTGT
ATCTAGGAGGTAACTAGCTTGCTTTTACAGGCTTGTAAGTGGAAGGGACTTGCCTTGTCTCAGATGAGACTTTGGACTGTGGACTT
TTGGGTTAATGCTGAAATGAGTTAAGACTTTGGGGAACTGTTGGGAAGGAATGATTGGTTTTGAAATGTGAGGATTATGAAATTTG
GAGGAGCCCAGGGAGGAATGATATGGTTTGGCTGTGTCCCCACCCAAATCTCAACTTGAATTGTATCTCCCAGAATTCCCACATGT
TGTGGGAGGGACGCAGTGGGAGGTAATTGAATCATGGAGGCCAGTCTTTTCTGTGCTATTCTCGTGATAATAAGTCCCACAAGATC
TGGTGGGTTTATGAAGAGTTTCTGCTTTTGCTTCTTCCTCATTTTGCTCTTGATGCCACCACGTAAGAAGTACCTTTTGCCTCCCA
CCTGATTTTGAGGCCTCCCCAGCCATGTGGAACTGTAAGTCCAATTAAA

Primer binding sites for amplification of ENTPD1-AS1 splice variants analyzed in this study

FANA antisense oligonucleotide target regions in *ENTPD1-AS1* splice variants.

GRCh38.p13 (Genome Reference Consortium Human Build 38)

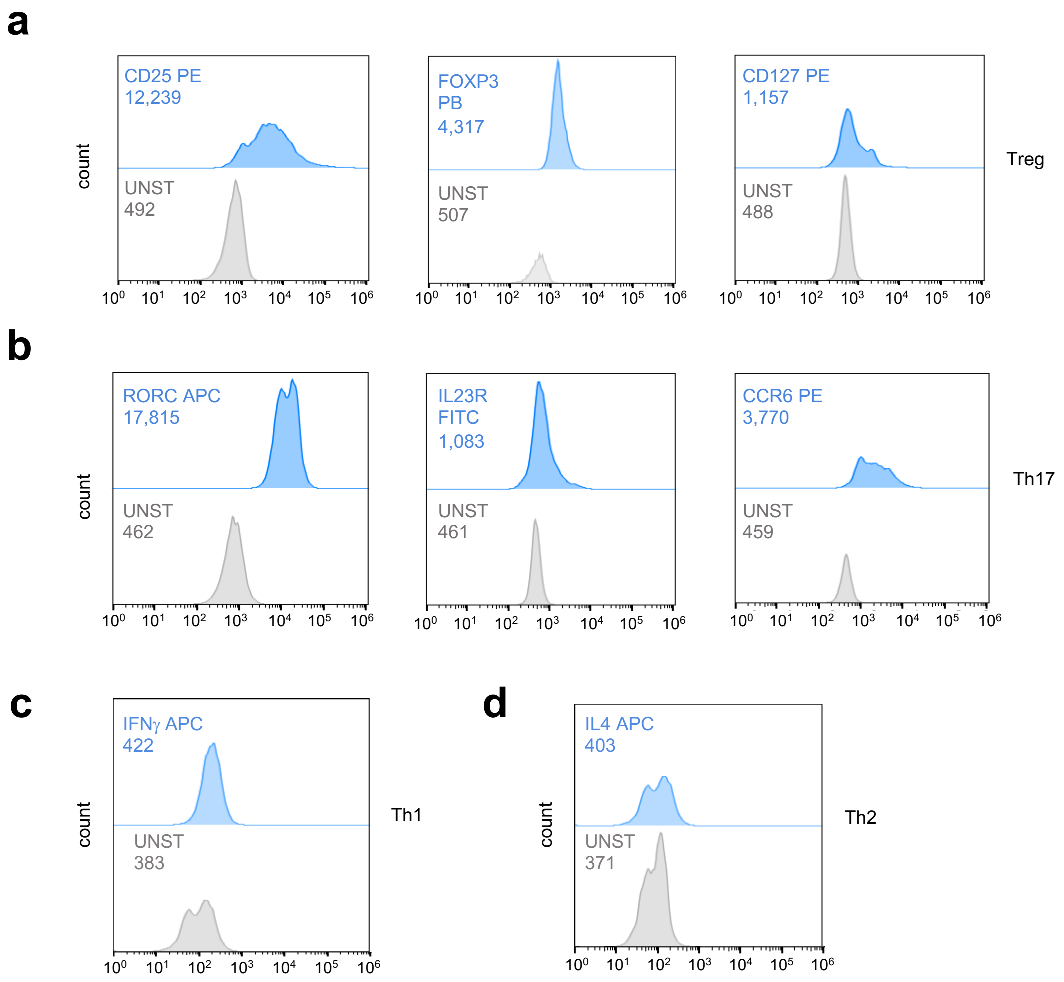

Supplementary Fig. 1. Polarization of peripheral blood derived Treg, Th17, Th1 and Th2 cells. Treg, Th17, Th1 and Th2 cells were polarized from circulating CD4 lymphocytes upon exposure to IL2, TGFβ and Dynabeads Human T activator CD3/CD28 (Treg); IL6, IL1β and TGFβ (Th17); IL12 and anti-IL4 antibodies (Th1); and to IL4 and anti-IFNγ antibodies (Th2). **a-d** Representative histograms showing CD25, FOXP3 and CD127 MFI in Treg; RORC, IL23R and CCR6 in Th17 cells; IFNγ in Th1 lymphocytes; and IL4 in Th2 cells from one healthy subject are shown. MFI values are indicated within the histogram plots.

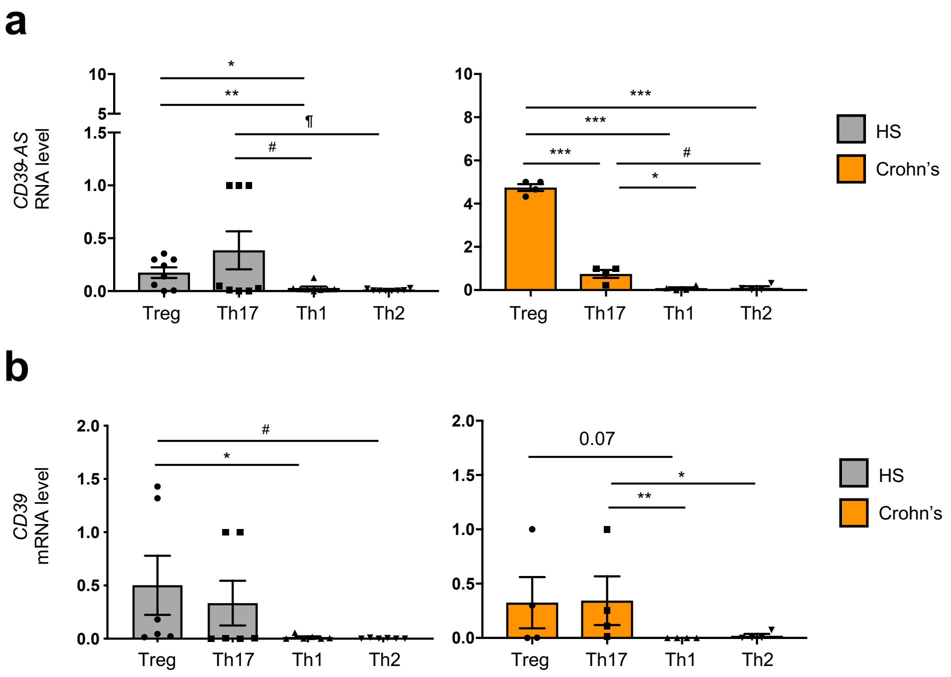

Supplementary Fig. 2. *CD39-AS* RNA levels in T cell subsets. Treg, Th17, Th1 and Th2 cells polarized from peripheral blood CD4 cells obtained from healthy subjects (HS) and Crohn's disease patients were tested for expression of *CD39-AS* RNA and *CD39* mRNA. **a-b** Mean ± SEM *CD39-AS* RNA and *CD39* mRNA levels in Treg, Th17, Th1 and Th2 cell subsets (*CD39-AS* RNA: n=8 HS and n=4 Crohn's disease patients; *CD39* mRNA: n=6 HS and n=4 Crohn's disease patients). Levels of *CD39-AS* RNA and *CD39* mRNA are higher in Treg and Th17 cells than in Th1 and Th2 subsets in both health and Crohn's disease (**a** *P=0.014, **P=0.005, $^{\#}$P=0.046, ¶P=0.037 for HS and *P=0.013, ***P<0.001, $^{\#}$P=0.018 for Crohn's patients using one-way ANOVA followed by Tukey's multiple comparisons test; **b** *P=0.013, $^{\#}$P=0.014 for HS and *P=0.032, **P=0.0016 for Crohn's patients using one-way ANOVA followed by Tukey's multiple comparisons test).

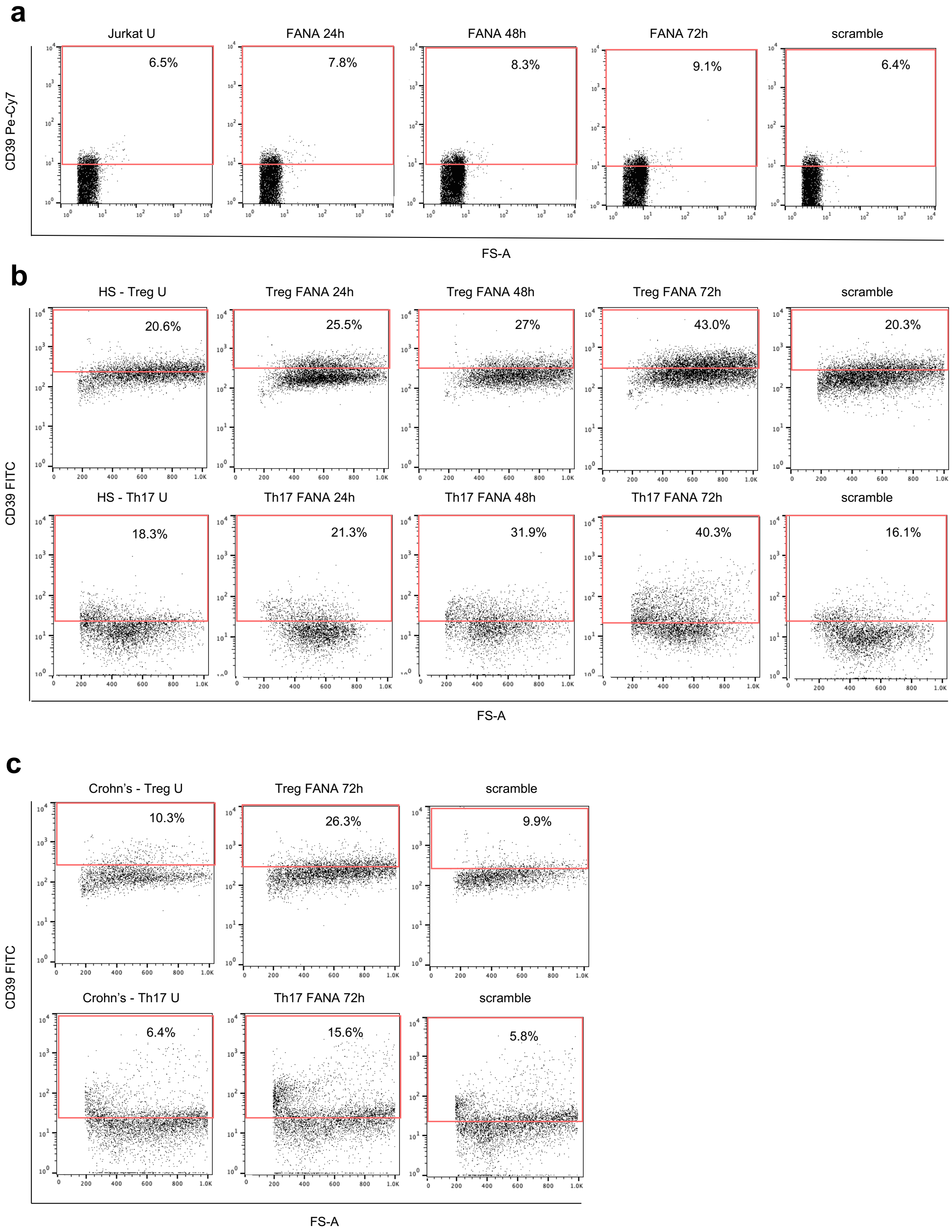

Supplementary Fig. 3. Effects of FANA-CD39-AS oligonucleotides on CD39$^+$ cell frequency. The frequency of CD39$^+$ cells was determined by flow cytometry in Jurkat, Treg and Th17 cells before and after treatment with FANA-CD39-AS oligonucleotides. **a** Frequency of CD39$^+$ cells is shown in untreated and FANA-CD39-AS oligonucleotide (or scramble) treated Jurkat cells at 24, 48 and 72 hours. Dot plots of forward scatter (FS-A) and CD39 fluorescence

are shown. **b** Dot plots of FS-A and CD39 fluorescence in untreated and FANA-CD39-AS oligonucleotide (or scramble) treated Treg and Th17 cells at 24, 48, 72 hours in one representative healthy subject (HS). **c** Dot plots of FS-A and CD39 fluorescence in untreated and FANA-CD39-AS oligonucleotide (or scramble) treated Treg and Th17 cells at 72 hours in one patient with Crohn's disease.

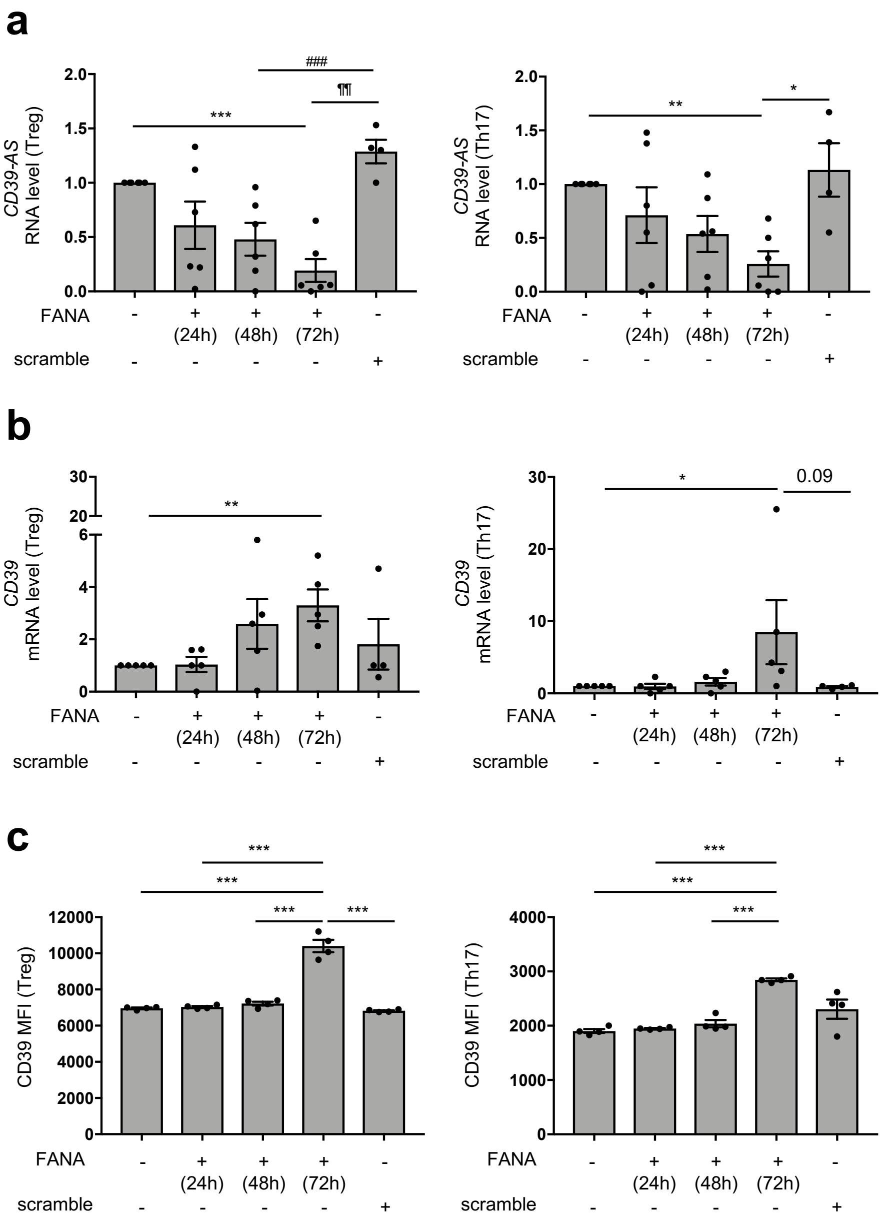

Supplementary Fig. 4. *CD39-AS* RNA silencing boosts *CD39* mRNA and MFI in Treg and Th17 cells. a-c Mean ± SEM *CD39-AS* RNA, *CD39* mRNA and CD39 MFI in Treg and Th17 cells before and after exposure to *CD39-AS* RNA silencing with FANA-CD39-AS oligonucleotides (or scramble) for 24, 48 and 72 hours (*CD39-AS* RNA: n=6 HS for untreated, FANA 24 hours, 48 hours and 72 hours; n=4 for scramble; *CD39* mRNA: n=5 HS for untreated, FANA 24 hours, 48 hours and 72 hours; n=4 for scramble; CD39 MFI: n=4 HS for all conditions) (**a** ***P=0.00056, $^{\#\#\#}$P=0.00013, ¶¶P=0.0046 for Treg and *P=0.03, **P=0.0014, for Th17; **b** **P=0.005, *P=0.017; **c** ***P<0.001 using one-way ANOVA followed by Tukey's multiple comparisons test).

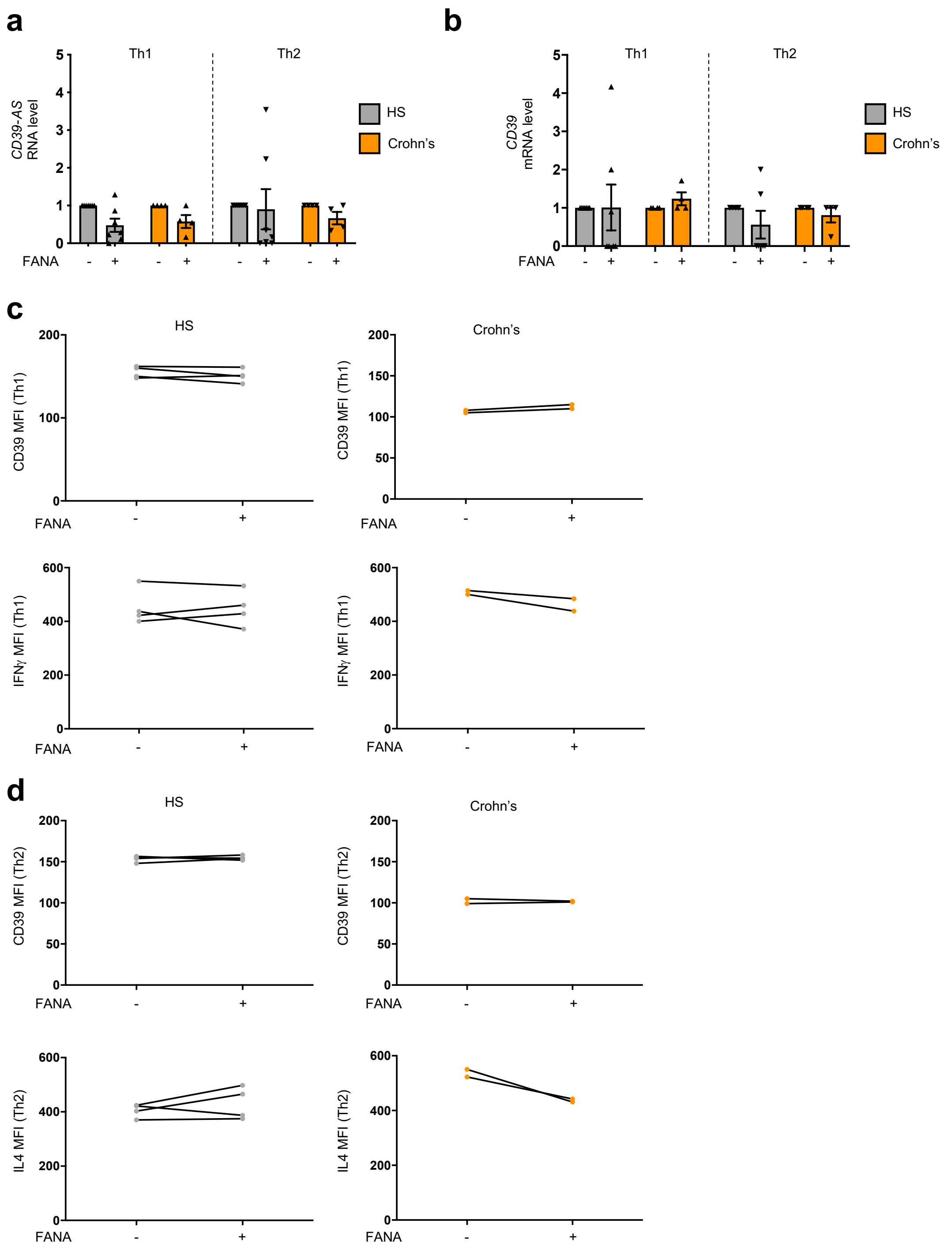

Supplementary Fig. 5. Effects of *CD39-AS* RNA silencing on Th1 and Th2 cells. Levels of *CD39-AS* RNA and *CD39* mRNA were measured in Th1 and Th2 cells, obtained upon polarization of CD4 cells from healthy subjects (HS) and patients with Crohn's disease. **a-b** Mean ± SEM *CD39-AS* RNA and *CD39* mRNA in Th1 and Th2 cells before and after exposure to FANA-CD39-AS oligonucleotides for 72 hours (*CD39-AS* RNA: n=7 HS and n=4 Crohn's disease patients; *CD39* mRNA: n=7 HS for Th1, n=6 HS for Th2 and n=4 Crohn's disease

patients for both cell types). **c-d** Effects of *CD39-AS* RNA silencing on CD39, IFNγ (Th1) and IL4 (Th2) were also assessed in 4 HS and in 2 Crohn's disease patients. No significant changes in any of these parameters were noted before and after cell exposure to FANA-CD39-AS oligonucleotides.

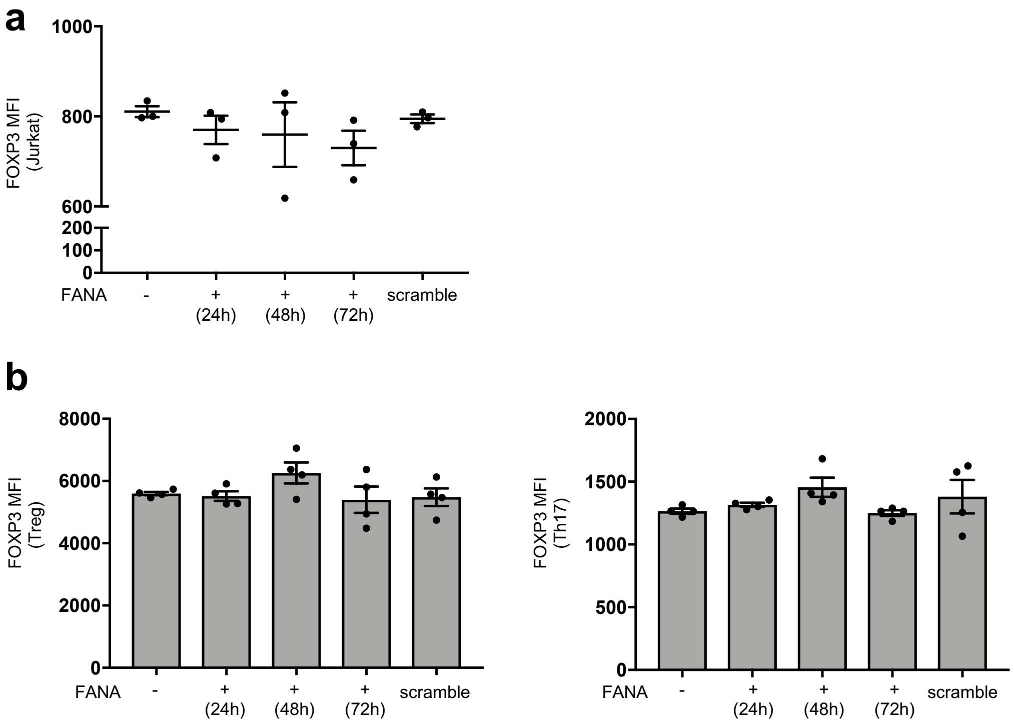

Supplementary Fig. 6. *CD39-AS* RNA silencing does not impact FOXP3 levels in Treg and Th17 cells. Expression of FOXP3 in Jurkat, Treg and Th17 cells, the latter differentiated from peripheral blood derived CD4 lymphocytes, was determined by flow cytometry and expressed as MFI. **a-b** Mean ± SEM FOXP3 MFI in untreated and FANA-CD39-AS oligonucleotide treated Jurkat (n=3 replicates), Treg and Th17 cells for 24, 48 and 72 hours (n=4 HS). Silencing of *CD39-AS* RNA did not change FOXP3 MFI in all cell types.

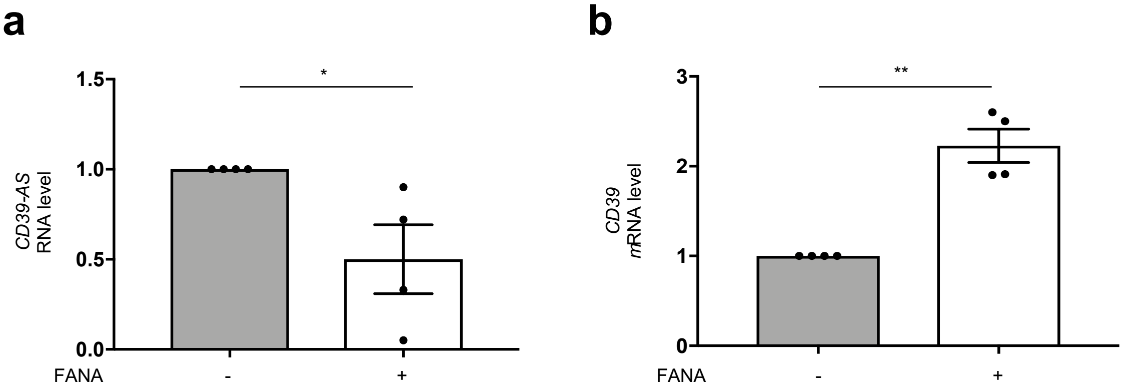

Supplementary Fig. 7. In vitro silencing of *CD39-AS* RNA in human CD4 cells. Human CD4 cells, obtained from one healthy blood donor were assessed for *CD39-AS* RNA and *CD39* mRNA levels before and after 72-hours treatment with FANA-CD39-AS oligonucleotides. **a-b** Mean ± SEM *CD39-AS* RNA and *CD39* mRNA levels following CD4 T cell exposure to 10 μM FANA-CD39-AS oligonucleotides for 72 hours (n=4 replicates) (**a** *P=0.039; **b** **P=0.007 using two-sided paired t test).

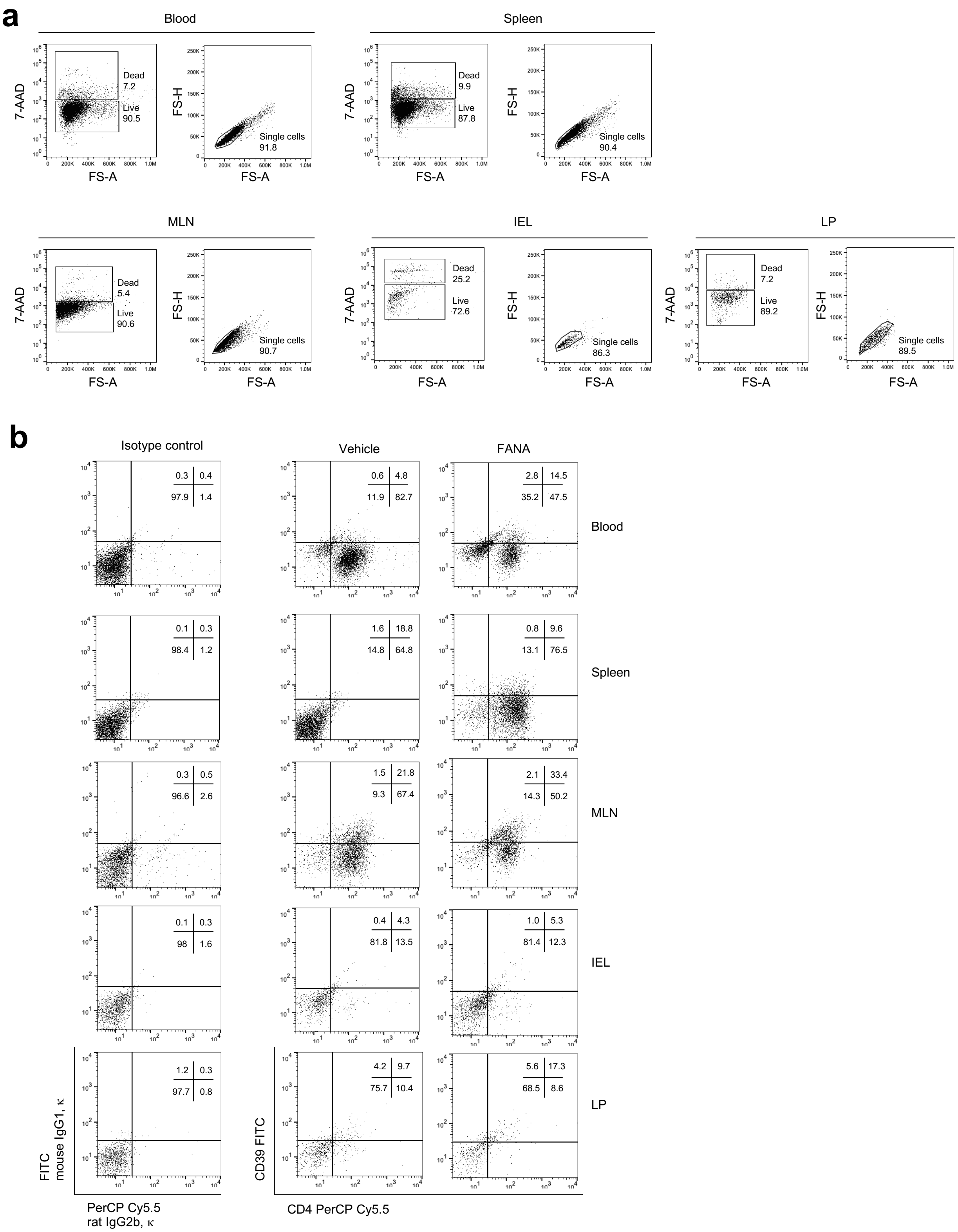
a
Blood
Spleen
7-AAD
FS-H
FS-A
Dead 7.2
Live 90.5
Single cells 91.8
Dead 9.9
Live 87.8
Single cells 90.4
MLN
IEL
LP
Dead 5.4
Live 90.6
Single cells 90.7
Dead 25.2
Live 72.6
Single cells 86.3
Dead 7.2
Live 89.2
Single cells 89.5
b
Isotype control
Vehicle
FANA
Blood
Spleen
MLN
IEL
LP
0.3 0.4 97.9 1.4
0.6 4.8 11.9 82.7
2.8 14.5 35.2 47.5
0.1 0.3 98.4 1.2
1.6 18.8 14.8 64.8
0.8 9.6 13.1 76.5
0.3 0.5 96.6 2.6
1.5 21.8 9.3 67.4
2.1 33.4 14.3 50.2
0.1 0.3 98 1.6
0.4 4.3 81.8 13.5
1.0 5.3 81.4 12.3
1.2 0.3 97.7 0.8
4.2 9.7 75.7 10.4
5.6 17.3 68.5 8.6
FITC mouse IgG1, κ
PerCP Cy5.5 rat IgG2b, κ
CD39 FITC
CD4 PerCP Cy5.5

c

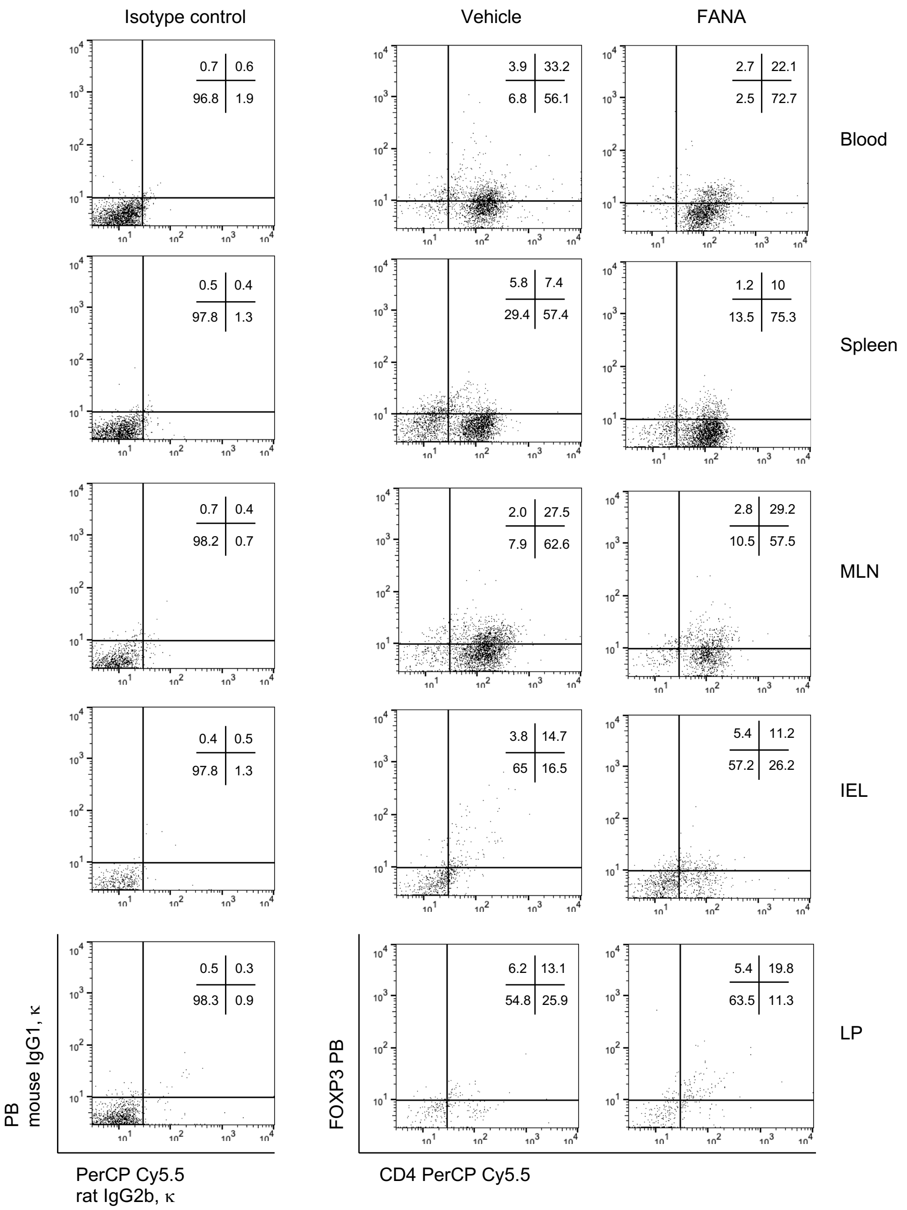
Isotype control
Vehicle
FANA
Blood
Spleen
MLN
IEL
LP
0.7 0.6 96.8 1.9
3.9 33.2 6.8 56.1
2.7 22.1 2.5 72.7
0.5 0.4 97.8 1.3
5.8 7.4 29.4 57.4
1.2 10 13.5 75.3
0.7 0.4 98.2 0.7
2.0 27.5 7.9 62.6
2.8 29.2 10.5 57.5
0.4 0.5 97.8 1.3
3.8 14.7 65 16.5
5.4 11.2 57.2 26.2
0.5 0.3 98.3 0.9
6.2 13.1 54.8 25.9
5.4 19.8 63.5 11.3
PB mouse IgG1, κ
FOXP3 PB
PerCP Cy5.5 rat IgG2b, κ
CD4 PerCP Cy5.5

d

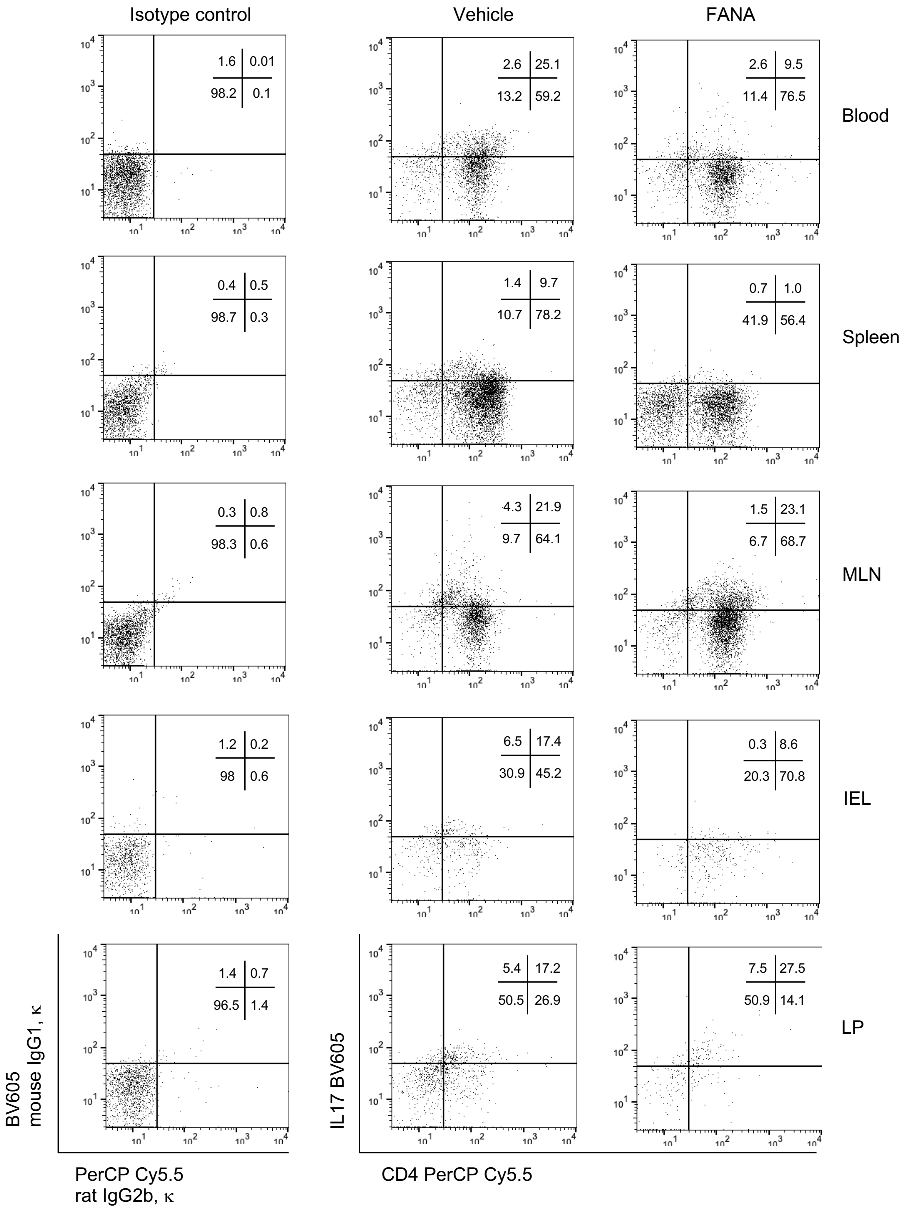
Isotype control
Vehicle
FANA
1.6 0.01 98.2 0.1
2.6 25.1 13.2 59.2
2.6 9.5 11.4 76.5
Blood
0.4 0.5 98.7 0.3
1.4 9.7 10.7 78.2
0.7 1.0 41.9 56.4
Spleen
0.3 0.8 98.3 0.6
4.3 21.9 9.7 64.1
1.5 23.1 6.7 68.7
MLN
1.2 0.2 98 0.6
6.5 17.4 30.9 45.2
0.3 8.6 20.3 70.8
IEL
1.4 0.7 96.5 1.4
5.4 17.2 50.5 26.9
7.5 27.5 50.9 14.1
LP
BV605 mouse IgG1, κ
PerCP Cy5.5 rat IgG2b, κ
IL17 BV605
CD4 PerCP Cy5.5

e

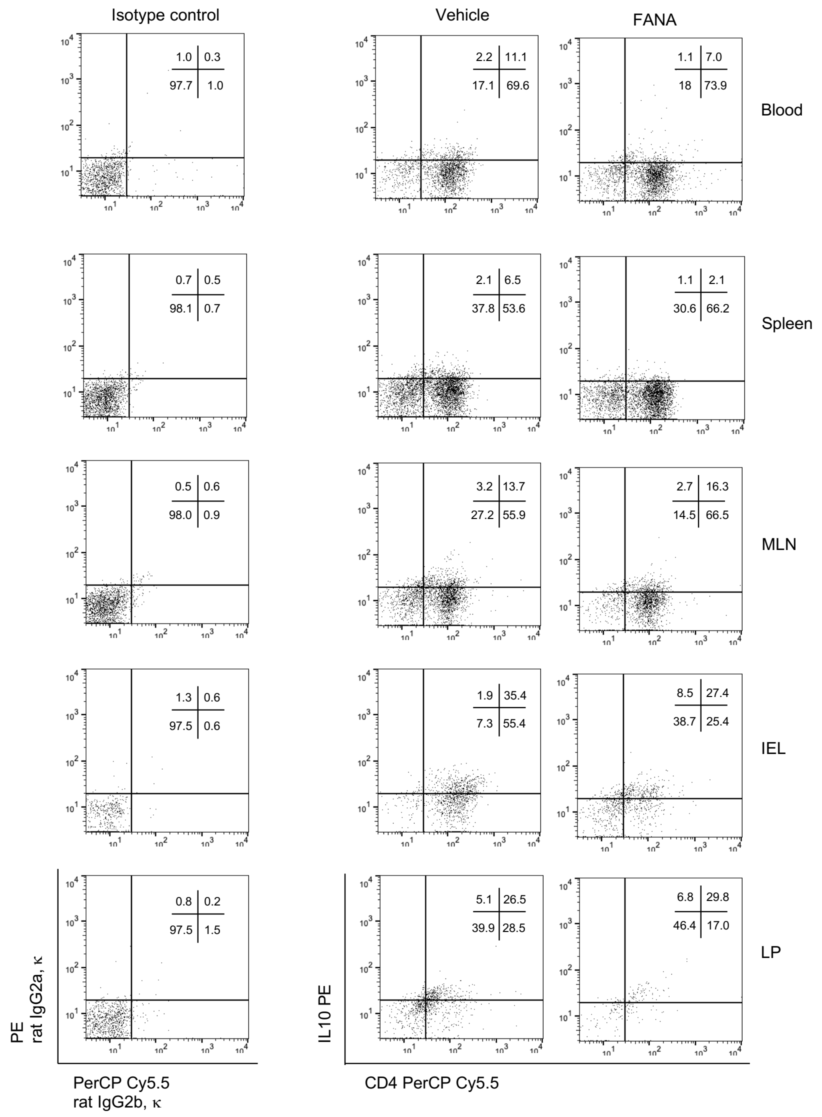

f

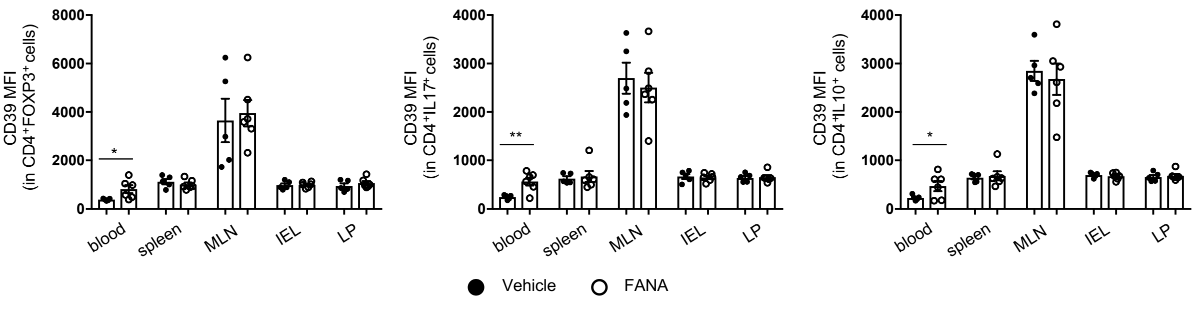

Supplementary Fig. 8. Effect of FANA-CD39-AS treatment on CD4 cell phenotype in TNBS induced colitis in humanized NOD/scid/gamma mice. Following reconstitution with healthy blood donor derived CD4 cells, NOD/scid/gamma mice were sensitized and administered

TNBS. At the same time of TNBS administration some animals were treated with FANA-CD39-AS oligonucleotides. Mice were harvested 72 hours later and frequency of human $CD4^{+}CD39^{+}$, $CD4^{+}FOXP3^{+}$, $CD4^{+}IL17^{+}$ and $CD4^{+}IL10^{+}$ cells within peripheral blood, spleen, mesenteric lymph node (MLN), intra-epithelial (IEL) and lamina propria (LP) derived lymphocytes was determined by flow cytometry. **a** Lymphocytes were initially stained with 7-AAD viability staining solution to exclude dead cells. Viable cells were then checked for the presence of doublets in the FS-A and FS-H flow cytometry plots. Single cells were gated and analysis carried on. Cell frequencies are indicated next to the gate. Representative FS-A and 7-AAD and FS-A and FS-H flow cytometry plots of peripheral blood, spleen, MLN, IEL and LP lymphocytes obtained from a representative vehicle and FANA-CD39-AS treated mice are shown. **b-e** Dot plots of isotype controls, human CD4 and CD39, FOXP3, IL17 and IL10 fluorescence in peripheral blood, spleen, MLN, IEL and LP lymphocytes in one representative vehicle and one FANA-CD39-AS treated mice are shown. Cell frequencies are shown in the upper right quadrant. **f** Mean ± SEM CD39 MFI in $CD4^{+}FOXP3^{+}$, $CD4^{+}IL17^{+}$ and $CD4^{+}IL10^{+}$ cells in different compartments in vehicle and FANA-CD39-AS treated mice. FANA-CD39-AS treatment increases CD39 MFI in all these cell populations in the peripheral blood (vehicle treated mice, n=5; FANA-CD39-AS treated mice, n=6) ($^{*}P=0.039$ for $CD4^{+}FOXP3^{+}$, $^{**}P=0.009$ for $CD4^{+}IL17^{+}$ and $^{*}P=0.047$ for $CD4^{+}IL10^{+}$ using two-sided unpaired t test).

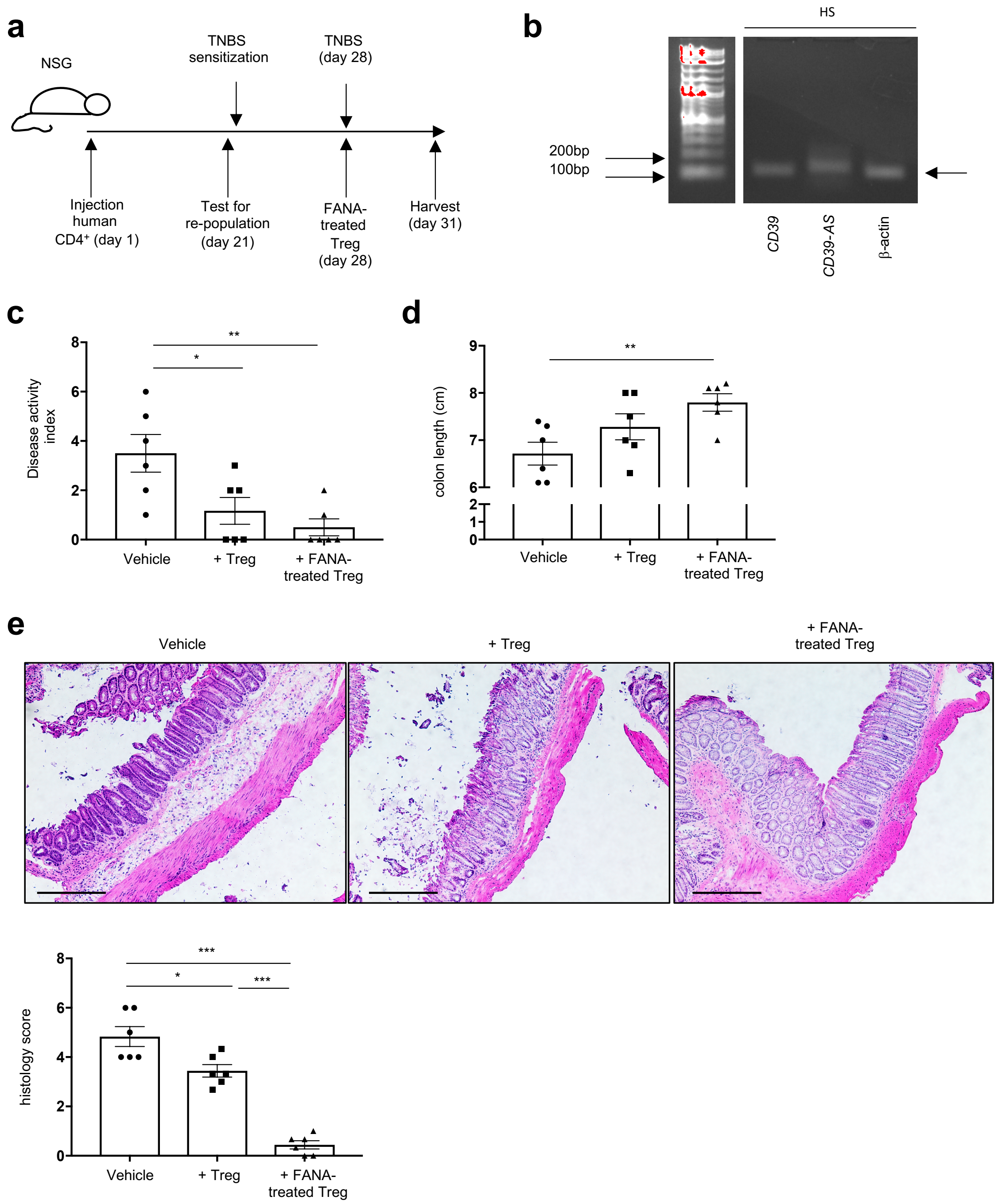

Supplementary Fig. 9. Silencing of *CD39-AS* RNA ameliorates Treg suppressive ability in TNBS induced experimental colitis in humanized NOD/scid/gamma mice. a NOD/scid/gamma female recipients were injected with CD4 cells, obtained from one healthy blood donor. After three weeks, mice were checked for human chimerism and those animals showing more than 10% human chimerism were sensitized to TNBS. One week later, mice were administered a single enema of TNBS and injected $CD4^{+}CD25^{high}CD127^{low}$ Treg, immunomagnetically sorted from the peripheral blood of the same donor and exposed to 10 μM FANA-CD39-AS oligonucleotides. Mice were sacrificed 72 hours later. **b** Cropped gel of RT-

PCR showing positivity for CD39 and *CD39-AS* RNA in healthy blood donor Treg prior to FANA-CD39-AS treatment (representative of three independent experiments with similar results). Full scan gel is provided in Source Data file. **c** Mean ± SEM disease activity index at the time of harvest in TNBS mice treated with vehicle (n=6), or with untreated (n=6) or FANA-CD39-AS oligonucleotide treated Treg (n=6) (*P=0.031, **P=0.0049 using one-way ANOVA followed by Tukey's multiple comparisons test). **d** Mean ± SEM colon length (cm) at the harvest (**P=0.0051 using one-way ANOVA followed by Tukey's multiple comparisons test). **e** Hematoxylin & Eosin staining of colon sections (original magnification, x10, scale bar: 200 μM). Mean ± SEM histology score at the time of harvesting is also shown (*P=0.014, ***P<0.001 using one-way ANOVA followed by Tukey's multiple comparisons test).

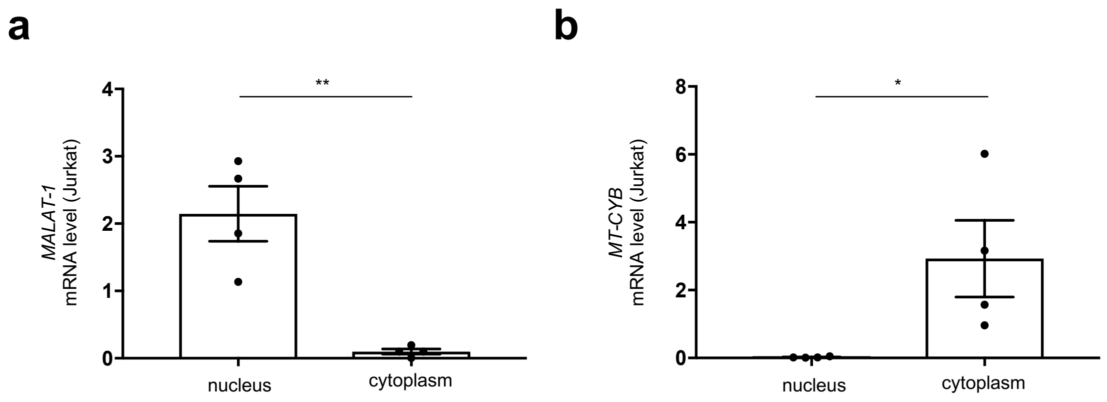

Supplementary Fig. 10. Validation of subcellular fractioning. Total RNA from nuclear and cytosolic subcellular fractions was obtained from Jurkat cells and validated for *MALAT1* and *MT-CYB* expression by RT-qPCR. **a-b** Mean ± SEM *MALAT-1* and *MT-CYB* mRNA levels in the nuclear and cytosolic fraction of Jurkat cells (n=4 replicates) (**a** **P=0.0024; **b** *P=0.042 using two-sided unpaired t test).

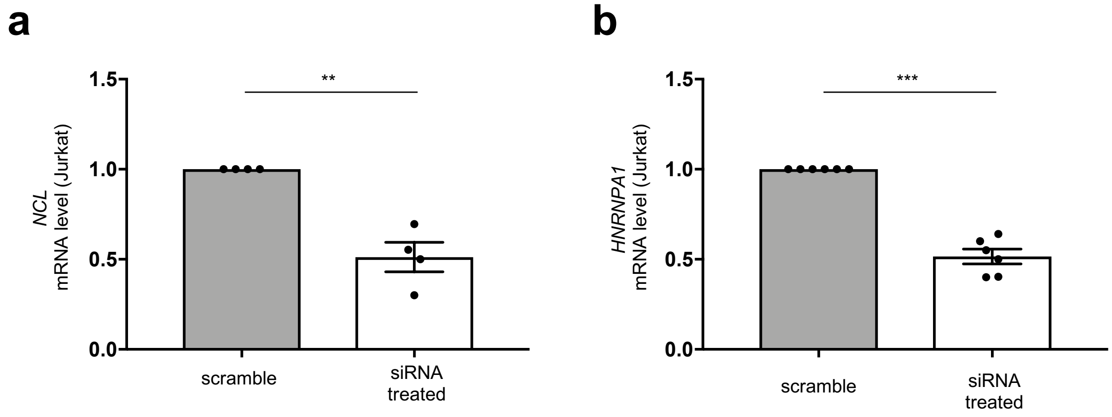

Supplementary Fig. 11. NCL and HNRNPA1 silencing via RNAi. Jurkat cells were treated with scramble or siRNA specific to NCL and HNRNPA1. Silencing was validated fourteen hours later via RT-qPCR analysis. **a-b** Mean ± SEM *NCL* and *HNRNPA1* mRNA levels in the absence or presence of specific siRNA in Jurkat cells (n=4 replicates for *NCL* and n=6 replicates for *HNRNPA1*) (**a** **P=0.009; **b** ***P<0.001 using two-sided unpaired t test).

Supplementary Table 1. Primers used for qPCR and RNA pulldown assays

	**Forward**	**Reverse**
CD39/ENTPD1-AS1 (qPCR)	5′ AATACTGTGACACTGACCACCAGG 3′	5′ TGAATCCACTCTGTCTCCTGCA 3′
CD39/ENTPD1 (qPCR)	5′ AGGTGCCTATGGCTGGATTAC 3′	5′ CCAAAGCTCCAAAGGTTTCCT 3′
MALAT-1 (qPCR)	5′ AAGCCCAAATCTCAAGCGGT 3′	5′ AAGTTGCTTGTGGGGAGACC 3′
MT-CYB (qPCR)	5′ ACCCCCTAGGAATCACCTCC 3′	5′ GCCTAGGAGGTCTGGTGAGA 3′
Nucleolin (qPCR)	5′ GATCACCTAATGCCAGAAGCCAGCCATCC 3′	5′ CAAAGCCGCCTCTGCCTCCACCAC 3′
HNRNPA1 (qPCR)	5′ AAGCAATTTTGGAGGTGGTG 3′	5′ ATAGCCACCTTGGTTTCGTG 3′
ENST00000452728.5 (RNA pulldown)	5′ TAATACGACTCACTATAGGGCTGTCATGGCTGCTGTCAGTGA 3′	5′ TTTACTTCAGTTCCCAACAAGGTCTTC 3′
ENST00000414006.2 (RNA pulldown)	5′ TAATACGACTCACTATAGGGCACGCGCCTTTGGGAATCTTC 3′	5′ TATCTTCTTCTGAGCCCTCCAGATT 3′

Sequence of forward and reverse primer pairs used for qPCR and RNA pulldown (template amplification) experiments are shown.

Orange underlined sequence represents the T7 promoter region.

Supplementary Table 2. Total spectrum counts - *ENTPD1-AS1-209* (*ENST00000452728.5*) variant (v1)

Identified Proteins	Total spectrum counts	Accession Number	Alternate ID	Molecular Weight
Nucleolin	21	NUCL_HUMAN	NCL	77 kDa
Heterogeneous nuclear ribonucleoprotein A1	19	ROA1_HUMAN	HNRNPA1	39 kDa
Heterogeneous nuclear ribonucleoproteins A2/B1	17	ROA2_HUMAN	HNRNPA2B1	37 kDa
Insulin-like growth factor 2 mRNA-binding protein 1	12	IF2B1_HUMAN	IGF2BP1	63 kDa
RNA-binding motif protein, X chromosome	9	RBMX_HUMAN	RBMX	42 kDa
Heterogeneous nuclear ribonucleoprotein A3	9	ROA3_HUMAN	HNRNPA3	40 kDa
Heterogeneous nuclear ribonucleoprotein M	8	HNRPM_HUMAN	HNRNPM	78 kDa
Ras GTPase-activating protein-binding protein 1	8	G3BP2_HUMAN	G3BP2	54 kDa
Heterogeneous nuclear ribonucleoprotein A0	8	ROA0_HUMAN	HNRNPA0	31 kDa
IFN-inducible ds RNA-dependent PK activator A	7	PRKRA_HUMAN	PRKRA	34 kDa
40S ribosomal protein S19	6	RS19_HUMAN	RPS19	16 kDa
Heterogeneous nuclear ribonucleoprotein L	6	HNRPL_HUMAN	HNRNPL	64 kDa
Insulin-like growth factor 2 mRNA-binding protein 3	6	IF2B1_HUMAN	IGF2BP1	63 kDa
Single-stranded DNA-binding protein, mitochondrial	5	SSBP_HUMAN	SSBP1	17 kDa
Nuclease-sensitive element-binding protein 1	4	YBOX1_HUMAN	YBX1	36 kDa
Synaptotagmin-like protein 4	4	SYTL4_HUMAN	SYTL4	76 kDa
Putative 40S ribosomal protein S10-like	4	RS10L_HUMAN (+1)	RPS10P5	20 kDa
Non-POU domain-containing octamer-binding protein	4	NONO_HUMAN	NONO	54 kDa
Replication protein A 32 kDa subunit	4	RFA2_HUMAN	RPA2	29 kDa
Caprin-1	3	CAPR1_HUMAN	CAPRIN1	78 kDa
Ras GTPase-activating protein-binding protein 2	3	G3BP2_HUMAN	G3BP2	54 kDa
Y-box-binding protein 3	3	YBOX3_HUMAN	YBX3	40 kDa
THO complex subunit 4	2	THOC4_HUMAN	ALYREF	27 kDa
Double-stranded RNA-specific adenosine deaminase	2	DSRAD_HUMAN	ADAR	136 kDa
40S ribosomal protein S14	2	RS14_HUMAN	RPS14	16 kDa
Elongation factor 1-alpha 1	2	EF1A1_HUMAN (+1)	EEF1A1	50 kDa

Supplementary Table 3. Total spectrum counts - *ENTPD1-AS1-201 (ENST00000414006.2)* variant (v2)

Identified Proteins	Total spectrum counts	Accession Number	Alternate ID	Molecular Weight
Nucleolin	14	NUCL_HUMAN	NCL	77 kDa
Heterogeneous nuclear ribonucleoprotein A1	7	ROA1_HUMAN	HNRNPA1	39 kDa
IFN-inducible ds RNA-dependent PK activator A	4	PRKRA_HUMAN	PRKRA	34 kDa
Heterogeneous nuclear ribonucleoprotein M	3	HNRPM_HUMAN	HNRNPM	78 kDa
Single-stranded DNA-binding protein, mitochondrial	3	SSBP_HUMAN	SSBP1	17 kDa
Synaptotagmin-like protein 4	3	SYTL4_HUMAN	SYTL4	76 kDa
RNA-binding motif protein, X chromosome	2	RBMX_HUMAN	RBMX	42 kDa
Heterogeneous nuclear ribonucleoproteins A2/B1	2	ROA2_HUMAN	HNRNPA2B1	37 kDa
40S ribosomal protein S19	2	RS19_HUMAN	RPS19	16 kDa
Nuclease-sensitive element-binding protein 1	2	YBOX1_HUMAN	YBX1	36 kDa
Insulin-like growth factor 2 mRNA-binding protein 3	2	IF2B3_HUMAN	IGF2BP3	64 kDa
THO complex subunit 4	2	THOC4_HUMAN	ALYREF	27 kDa
Putative 40S ribosomal protein S10-like	2	RS10L_HUMAN (+1)	RPS10P5	20 kDa
Caprin-1	2	CAPR1_HUMAN	CAPRIN1	78 kDa
Insulin-like growth factor 2 mRNA-binding protein 1	1	IF2B3_HUMAN	IGF2BP3	64 kDa
Heterogeneous nuclear ribonucleoprotein A3	1	ROA3_HUMAN	HNRNPA3	40 kDa
Heterogeneous nuclear ribonucleoprotein L	1	HNRPL_HUMAN	HNRNPL	64 kDa
Heterogeneous nuclear ribonucleoprotein A0	1	ROA0_HUMAN	HNRNPA0	31 kDa
Non-POU domain-containing octamer-binding protein	1	NONO_HUMAN	NONO	54 kDa
Ras GTPase-activating protein-binding protein 1	0.01	G3BP2_HUMAN	G3BP2	54 kDa
Double-stranded RNA-specific adenosine deaminase	0.01	DSRAD_HUMAN	ADAR	136 kDa
Replication protein A 32 kDa subunit	0.01	RFA2_HUMAN	RPA2	29 kDa
Ras GTPase-activating protein-binding protein 2	0.01	G3BP2_HUMAN	G3BP2	54 kDa
Y-box-binding protein 3	0.01	YBOX3_HUMAN	YBX3	40 kDa
40S ribosomal protein S14	0.01	RS19_HUMAN	RPS19	16 kDa
Elongation factor 1-alpha 1	0.01	EF1A1_HUMAN (+1)	EEF1A1	50 kDa

Supplementary Table 4. Demographic and clinical data.

Crohn's Disease (n=70)	
Active/Inactive	30/40
Sex (F/M)	32/38
Montreal Age	'1' n=1<br>'2' n=39<br>'3' n=30
Montreal Type	'1' n=47<br>'2' n=10<br>'3' n=13
HBI<br>range (median)	1-12 (3)
Infliximab	31
Steroids	16
Mercaptopurine	8

F: female

M: male

Montreal Age: '1' < 16 years; '2' 17-40 years; '3' > 40 years

Montreal Type: '1' inflammatory; '2' stricturing; '3' penetrating

HBI: Harvey Bradshaw Index
