# Supplementary material for: Endogenous antisense RNA curbs CD39 expression in Crohn’s disease
Source: Nat Commun. 2020 Nov 18;11:5894. doi: 10.1038/s41467-020-19692-y (PMC7676266; doi:10.1038/s41467-020-19692-y)
Supplement: Supplementary file 3 — Reporting Summary [file 41467_2020_19692_MOESM3_ESM.pdf]

nature research

Corresponding author(s): Maria Serena Longhi

Last updated by author(s): Sep 30, 2020

Reporting Summary

Nature Research wishes to improve the reproducibility of the work that we publish. This form provides structure for consistency and transparency in reporting. For further information on Nature Research policies, see our Editorial Policies and the Editorial Policy Checklist.

Statistics

For all statistical analyses, confirm that the following items are present in the figure legend, table legend, main text, or Methods section.

n/a	Confirmed	
☐	☒	The exact sample size (*n*) for each experimental group/condition, given as a discrete number and unit of measurement
☐	☒	A statement on whether measurements were taken from distinct samples or whether the same sample was measured repeatedly
☐	☒	The statistical test(s) used AND whether they are one- or two-sided<br>*Only common tests should be described solely by name; describe more complex techniques in the Methods section.*
☒	☐	A description of all covariates tested
☒	☐	A description of any assumptions or corrections, such as tests of normality and adjustment for multiple comparisons
☐	☒	A full description of the statistical parameters including central tendency (e.g. means) or other basic estimates (e.g. regression coefficient) AND variation (e.g. standard deviation) or associated estimates of uncertainty (e.g. confidence intervals)
☐	☒	For null hypothesis testing, the test statistic (e.g. *F*, *t*, *r*) with confidence intervals, effect sizes, degrees of freedom and *P* value noted<br>*Give P values as exact values whenever suitable.*
☒	☐	For Bayesian analysis, information on the choice of priors and Markov chain Monte Carlo settings
☒	☐	For hierarchical and complex designs, identification of the appropriate level for tests and full reporting of outcomes
☐	☒	Estimates of effect sizes (e.g. Cohen's *d*, Pearson's *r*), indicating how they were calculated

Our web collection on statistics for biologists contains articles on many of the points above.

Software and code

Policy information about availability of computer code

Data collection	Data were collected using Microsft Excel for Mac (version 16.16.22)
Data analysis	Statistical analysis was performed using SPSS version 22 and GraphPad Prism, version 7.0e. Flow cytometry data were analyzed using FlowJo 2 software (version 10, TreeStar)

For manuscripts utilizing custom algorithms or software that are central to the research but not yet described in published literature, software must be made available to editors and reviewers. We strongly encourage code deposition in a community repository (e.g. GitHub). See the Nature Research guidelines for submitting code & software for further information.

Data

Policy information about availability of data

All manuscripts must include a data availability statement. This statement should provide the following information, where applicable:

- Accession codes, unique identifiers, or web links for publicly available datasets
- A list of figures that have associated raw data
- A description of any restrictions on data availability

All data that have been generated in this study are included in the Source Data file. For further request, please contact the corresponding Author.

Field-specific reporting

April 2020

Life sciences study design

All studies must disclose on these points even when the disclosure is negative.

Sample size	The sample size was determined on the basis of pilot studies that enabled to observe differences in variable means consistent with our previously published reports on human CD39 expression in T cells (pilot studies conducted using a two-sided 5% type 1 error; power calculated using GraphPad Stat Mate 2).
Data exclusions	No data were excluded from analysis. All the data that have been generated in this study have been included in the Source Data file.
Replication	Each experiment was successfully replicated at least three times. Replicate experiments gave consistent results.
Randomization	Allocation of subjects either to the study (Crohn's disease) or control group (healthy subjects) was made according to the diagnostic criteria for Crohn's disease. Allocation of animals to treatment or vehicle group was random. When using cell lines, wells that served as controls and those subjected to treatment were randomly chosen.
Blinding	In both animal and in vitro experiments using human derived cells and cell lines, investigators were blinded to the treatment of different experimental groups during data collection.

Reporting for specific materials, systems and methods

We require information from authors about some types of materials, experimental systems and methods used in many studies. Here, indicate whether each material, system or method listed is relevant to your study. If you are not sure if a list item applies to your research, read the appropriate section before selecting a response.

Materials & experimental systems

n/a	Involved in the study	
☐	☒	Antibodies
☐	☒	Eukaryotic cell lines
☒	☐	Palaeontology and archaeology
☐	☒	Animals and other organisms
☐	☒	Human research participants
☐	☒	Clinical data
☒	☐	Dual use research of concern

Methods

n/a	Involved in the study	
☒	☐	ChIP-seq
☐	☒	Flow cytometry
☒	☐	MRI-based neuroimaging

Antibodies

Antibodies used	Antibodies for flow cytometry: anti-human CD4 (clone # OKT4, Biolegend, cat. # 317414, lot # B163309), CD25 (clone # M-A251, Biolegend, cat. # 356134, lot # B186553), CD127 (clone # A019D5, Biolegend, cat. # 351303, lot # B143868), CD39 (clone # A1, Biolegend, cat. # 328206, lot # B171237), CCR6 (clone # G034E3, Biolegend, cat. # 353409, lot # B148950), IL23 receptor (clone # 218213, R&D Systems, cat. # FAB14001F, lot # XZM0208101), FOXP3 (clone # PCH101, eBioscience, cat. # 12-4776-42), RORC (clone # AFKJS-9, eBioscience, cat. # 17-6988-82, lot # 2010688), IFNgamma (clone # 4S.B3, Biolegend, cat. # 502511, lot # B145646), IL4 (clone # 8D4-8, Biolegend, cat. # 500704, lot # B150137), CD4 (clone # A161A1, Biolegend, cat. # 357414, lot # B29135), FOXP3 (clone # 206D, Biolegend, cat. # 320108, lot # B244884), IL17 (clone # BL168, Biolegend, cat. # 512326, lot # B272369), IL10 (clone # JES3-19F1, Biolegend, cat. # 506804, lot # B263814), IFNgamma (clone # B27, BD Pharmingen, cat. # 557995, lot # 3288828), IgG2b, k isotype control (clone # RTK4530, Biolegend, cat. # 400631), mouse IgG1, k isotype control (clone # MOPC-21, Biolegend, cat. # 400107, # 400131, # 400161) and rat IgG2a, k isotype control (clone # RTK2758, Biolegend, cat. # 400507); 7-AAD viability staining solution (cat. # 420404, Biolegend, lot # B187843).<br>Neutralizing antibodies: anti-human IL4 antibodies (cat. # MAB204-SP, R&D Systems, lot # AVT0719091), anti-human IFNgamma antibodies (cat. # MAB285-SP, R&D Systems, lot # KW1919091).
Validation	All antibodies used for flow cytometry are commercially available and have been validated for species reactivity and application by the vendors; validation data are available in the vendors' website. Neutralizing antibodies used for cell differentiation were validated by the vendors (data available in the vendor's website) and further verified by us for inhibition properties using flow cytometry and ELISA in preliminary experiments.

Eukaryotic cell lines

Policy information about cell lines

Cell line source(s)	Jurkat, Raji, THP-1 and HCC1739BL cell lines were obtained from American Type Culture Collection (ATCC).
Authentication	The cell lines used in this study are commercially available through the vendor. Authentication was carried out by the vendor and cell phenotype verified by us by flow cytometry upon receipt.

Mycoplasma contamination	All cell lines were negative for Mycoplasma contamination.
Commonly misidentified lines (See ICLAC register)	No misidentified cell lines were used.

Animals and other organisms

Policy information about studies involving animals; ARRIVE guidelines recommended for reporting animal research

Laboratory animals	6-weeks old NOD/scid/gamma female mice were used. These were purchased from The Jackson Laboratory. Animals were housed under pathogen free conditions, at 21-23C, with a 12:12 hour dark-light cycle and relative humidity ranging from 45% to 55%.
Wild animals	No wild animals were used in the study.
Field-collected samples	No field-collected samples were used in the study.
Ethics oversight	Animal Care and Use Committee at Beth Israel Deaconess Medical Center (BIDMC), Boston, MA, USA.

Note that full information on the approval of the study protocol must also be provided in the manuscript.

Human research participants

Policy information about studies involving human research participants

Population characteristics	Patients with Crohn's disease: female/male: 32/38; Montreal age classification: 1 subject was less than 16 years old, 39 were between 17 and 40 years old, while 30 were over 40 years old; 31 subjects were on Infliximab, 16 were on steroids while 8 were on mercaptopurine. Controls were healthy blood donors (age and gender matched).
Recruitment	Patients were recruited from the Gastroenterology Division, Beth Israel Deaconess Medical Center (BIDMC), Boston, MA. Controls were healthy blood donors (Blood Donor Center at Children's Hospital, Boston, MA). No self-selection bias were present during recruitment of human participants.
Ethics oversight	IRB approval was granted by the Committee on Clinical Investigations, Beth Israel Deaconess Medical Center, Boston, MA (protocol # 2011P000202). Written informed consent was obtained from all study participants or legally authorized representatives.

Note that full information on the approval of the study protocol must also be provided in the manuscript.

Clinical data

Policy information about clinical studies

All manuscripts should comply with the ICMJEguidelines for publication of clinical research and a completedCONSORT checklist must be included with all submissions.

Clinical trial registration	N/A
Study protocol	N/A
Data collection	Clinical data were collected from hospital medical records at the time of patients' visit or admission to hospital.
Outcomes	N/A

Flow Cytometry

Plots

Confirm that:

- [x] The axis labels state the marker and fluorochrome used (e.g. CD4-FITC).
- [x] The axis scales are clearly visible. Include numbers along axes only for bottom left plot of group (a 'group' is an analysis of identical markers).
- [x] All plots are contour plots with outliers or pseudocolor plots.
- [x] A numerical value for number of cells or percentage (with statistics) is provided.

Methodology

Sample preparation	Cells were derived from peripheral blood and lamina propria CD4 cells, polarized into Treg, Th17, Th1 and Th2 cells and then analyzed by FACS.
Instrument	BD LSRII (BD Biosciences)
Software	FlowJo 2 software (version 10, TreeStar, Ashland, OR).

Cell population abundance	Frequency of polarized cells varied from 10-15% across the samples. Purity of CD4 cells was consistently above 92%. Purity of cell population was determined by FACS analysis and, in the case of Tregs, confirmed by functional assays.
Gating strategy	Live cells were gated after exclusion of dead cells and doublets. Positively stained cell populations were gated based on unstained, single stained and relevant isotype controls. Fluorescence compensation was adjusted based on fluorescence-minus-one method.

☒ Tick this box to confirm that a figure exemplifying the gating strategy is provided in the Supplementary Information.
